# Defamation lawsuits: academic sword or shield?

**DOI:** 10.15252/emmm.201708489

**Published:** 2017-10-16

**Authors:** Elizabeth Hall‐Lipsy, Sarah Malanga

**Affiliations:** ^1^ College of Pharmacy University of Arizona Tucson AZ USA; ^2^ Regulatory Science Fellow College of Law University of Arizona Tucson AZ USA

## Abstract

Scientists and academics are used to defending their theories, methods, and results in the classroom, at conferences, and in peer‐reviewed publications. But Pieter Cohen, an assistant professor at Harvard Medical School, learned that he had to defend his research in a defamation suit while reading a supplement industry trade publication (Robins, 2017). Cohen is known as a “dogged detective” for scrutinizing dietary supplements and for advocating for their stricter oversight by the US Food and Drug Administration (FDA).

Hi‐Tech Pharmaceuticals, a manufacturer of dietary supplements in Norcross, GA, sued Cohen and his three co‐authors based on a peer‐reviewed, scientific article about the chemical compositions of 21 weight‐loss supplements labeled as containing “*Acacia rigidula*”, a shrub native to Texas (Couzin‐Frankel, 2015; Robins, 2017; https://dockets.justia.com/docket/georgia/gandce/1:2015cv01413/215592). Cohen *et al* found that 11 of the 21 supplements tested—including those manufactured by Hi‐Tech—contained high doses of BMPEA, an amphetamine isomer, which had been synthesized in the 1930s as a potential amphetamine alternative (Cohen *et al*, 2016). Cohen's attorneys, hired by his employer, Harvard Medical School, were first successful in dismissing the lawsuit for lack of jurisdiction, since none of the authors had any contacts within the state of Georgia (Robins, 2017). Hi‐Tech then refiled its suit in Massachusetts, dropping the other two authors and reducing the requested damages. The company alleged libel, slander, product disparagement, and a violation of Massachusetts’ consumer protection law (Hi‐Tech Pharm., Inc. v. Cohen, 2016a,b).

In order to make a case for defamation, Hi‐Tech had to prove that (i) the defendant (Cohen) had made a statement, concerning plaintiff (Hi‐Tech) to a third party; (ii) the statement could damage the plaintiff's reputation in the community; (iii) the defendant was at fault in making the statement; and (iv) the statement either caused the plaintiff economic loss or is actionable without economic loss (Hi‐Tech Pharm., Inc. v. Cohen, 2016b). Cohen's attorneys moved to dismiss this second suit, alleging that his statements were not defamatory and that Hi‐Tech had failed to demonstrate that Cohen acted with the required level of culpability. Ultimately, the judge ruled that, under the 7th Amendment to the US Constitution, Hi‐Tech was entitled to a jury trial on their claims as there were material issues of fact for the jury to determine (Hi‐Tech Pharm., Inc. v. Cohen, 2016b). Within almost 6 months of filing the suit, the case proceeded to a jury trial, which lasted 7 days and resulted in a verdict in favor of Cohen and an award of his costs (not including attorneys’ fees) in the amount of US$7,178.24 (United States District Court, District of Massachusetts, 2017).

This would seem a successful outcome; yet, it came at significant personal cost for Cohen. Since the suit arose out of his employment as a faculty member, Harvard Medical School stepped in to defend him. But the attorneys were hired under Harvard's insurance and the insurer had agreed to cover only up to US$5 million in damages—at the time of filing the suit, Hi‐Tech's demands were much higher (Robins, 2017). In addition to the financial burden and uncertainties, Cohen lost valuable time and energy, time that he could have devoted to his research. Additionally, as required during the lawsuit, he had to provide Hi‐Tech with copies of his research materials and notes as well as his correspondence with the publisher and his colleagues about his research in question. While ultimately unsuccessful in court, Hi‐Tech's lawsuit effectively sent a warning to other academics that publishing and promoting their research could result in defending their work in expensive, time‐consuming legal action. In fact, Hi‐Tech's CEO is attributed with a comment that he “hope[s] that the long and costly legal battle will scare away other academics from investigating the supplement industry” (Robins, 2017).

In an almost reverse instance of this case, an academic used defamation as a sword. In 2008, *Nature* published an article “Self‐publishing editor set to retire”. It detailed how the editor of a theoretical‐physics journal, Mohamed El Naschie, was facing growing criticism that he had used the journal to publish his own papers: During the previous year, 60 of his papers had appeared in the journal (Schiermeier, 2008). The *Nature* article quoted several scientists, one who commented that “it's plain obvious that there was either zero or at best very poor, peer review of [El Naschie's] own papers” (Schiermeier, 2008). Moreover, the article noted the high impact factor of the journal for 2007, which may have been “the result of a high rate of self‐citation”. During that year, the journal had published 31 papers not written by El Naschie, which included 58 citations to his self‐published work (Schiermeier, 2008).

El Naschie sued MacMillan, the then publisher of *Nature,* and the author of the article, Quirin Schiermeier, in the UK for libel and other claims, which were dismissed in an earlier trial. Under existing law, the article was indisputably defamatory toward El Naschie, but almost three and a half years after it was published, a British court ruled in favor of *Nature* and Schiermeier, finding that the article was “substantially true […] that it contains comments which are defensible as honest comment, and that it was the product of responsible journalism…” (El Naschie v. Macmillan Publishers Ltd, 2012). While the defendants were awarded the costs of the action, it is unlikely that any money was ever recovered from El Naschie. Schiermeier later acknowledged that he was fortunate that Nature Publishing Group had the resources, opportunity, and interest in vigorously defending him, but he related that the experience was personally very taxing and distressing (Schiermeier, 2012).

There is a key similarity in the two cases: In both instances, the defendants were supported by their employers who had the financial resources and commitment to protect the publication. Not all scholars, researchers, or academics, however, can afford to defend a suit to its resolution, and not all scholars may be supported by their institutions. A wealthy, private institution, such as Harvard University, could be called upon to defend faculty members in defamation or libel lawsuits that arise out of scholarly and academic publications if this falls within the scope of a faculty member's employment. But public institutions of higher education, smaller private institutions, and other non‐profits may find that the burden of defending a controversial publication outweighs their interest in publishing new ideas, comments, theories, and commentaries. While advocacy groups may step in to protect free speech and attorneys may take cases on a *pro bono* basis, a lawsuit still incurs considerable time and expense that may exceed available resources. Moreover, while institutions and researchers are potentially liable for libel and defamation, so too are the journals in which the research is published. Since many scholarly journals exercise extensive editorial control and judgment, they too are potentially subject to defamation suits along with their authors.

There are obvious key differences between the two cases: In one instance, a researcher was sued for publishing his work in the USA; in the other, a researcher sued a scholarly journal for disparaging comments about his work in the UK. And while the USA and the UK often align in legal topics, their treatment of defamation law differs. At times, this has previously resulted in “libel tourism”, when a litigant brings a suit in a country or location where he or she is more likely to win because the laws are more favorable. In US courts, the person bringing a lawsuit for defamation/libel must bear the burden of proof; conversely, in UK courts, the defendant bears the burden of proof and must often prove the truth of any statement at issue. For example, after US author Rachel Ehrenfeld wrote a book about financing terrorism, a Saudi businessman whom she had accused of funding terrorism brought a case against her for libel in England. Her book was never published in the UK, and she did not appear or participate in the suit. The English court issued a default judgment requiring her to pay a penalty in the amount of US$250,000 and to destroy the book. Previously, a US court may have generally enforced such a foreign judgment against her, but in 2008, US Congress passed the Speech Act, which provides grounds for not recognizing a foreign defamation judgment unless the US court determines the foreign judgment is consistent with the First Amendment of the US Constitution.

In 2013, the UK responded to suits like El Naschie's by changing its defamation laws to reduce the potential for “libel tourism”. The Defamation Act changed the burden of proof from demonstrating that the public's estimation of the claimant would be lowered as a result of a comment, to now requiring a litigant to demonstrate that the comment caused harm or is likely to cause harm. The 2013 Act also provides for additional defenses: It codified a qualified privilege for a “publication on matters of public interest”—which was a successful defense for *Nature* and Schiermeier. Moreover, the Act includes a qualified privilege for statements in scientific or academic journals, if “the statement relates to a scientific or academic matter; and before the statement was published in the journal, an independent review of the statement's scientific or academic merit was carried out by (i) the editor of the journal, and (ii) one or more persons with expertise in the scientific or academic matter concerned” (Cohen, 2008). Thus, academic publications that meet the above requirements cannot be the subject of a defamation suit as long as the article is written without malice and is accurate. It is important to note that such an exception would not have applied to El Naschie's case and would not have barred the action against Nature Publishing Group and Schiermeier since it was not a scholarly article that met the qualified privilege requirements. Conversely, this defense would have applied to Cohen's case, but no such exception occurs under US defamation law.

More than half of the US states offer defenses against cases that are designed to prevent participation in public discourse. These are found under Anti‐Strategic Litigation Against Public Participation (SLAPP) laws and provide an affirmative defense to a lawsuit that is filed with the intent to censor, silence, or intimidate critics by burdening them with costly legal defenses. These Anti‐SLAPP laws allow the defendant to file a special motion to dismiss a claim for defamation that arises out of actions that are based on the defendant's right to exercise free speech, as protected under the US Constitution. But, much like UK defamation law, these Anti‐SLAPP laws can contribute to libel tourism as states provide different exceptions and apply only to certain types of petitioning behaviors, such as directly petitioning government entities or agencies (Simpson, 2016).

The Massachusetts Anti‐SLAPP statute, at issue in Hi‐Tech Pharmaceuticals vs. Cohen, limited petitioning activities to those addressed to legislative, executive, or judicial government bodies. In this instance, the judge agreed that Cohen's article was considered petitioning activity since he was urging the FDA to take action on certain supplements. The judge, however, found that Hi‐Tech had demonstrated a *prima facie* showing that Cohen's petitioning activities had no reasonable basis in fact or law and therefore concluded that dismissal of the case was premature given the plaintiff's right to trial by jury. In denying Cohen's Anti‐SLAPP motion, the judge did not mention that Cohen's publication was published after peer review, and neither was that issue emphasized in the Anti‐SLAPP pleadings. The court, however, did note that the case from commencement to trial took < 7 months (Hi‐Tech Pharm., Inc. v. Cohen, 2016b), which alleviated the burden on Cohen and that sanctions are available under Massachusetts laws if the lawsuit is determined to be without merit.

As a result of this case and others like it, there is renewed interest in a federal Anti‐SLAPP law. During the previous 8 years, various bills have been introduced in Congress, but none have made it out of congressional committee. However, libel laws and Anti‐SLAPP may receive greater attention given US President Donald Trump's comments that he is “going to open up our libel laws so when they write purposely negative and horrible and false articles, we can sue them and win lots of money” (Robins, 2017). While the President would have little opportunity to make changes to state laws, his words are reflective of the cases described above and carry a danger for academic publishing. Even if a libel lawsuit may ultimately be unsuccessful, its impact may still be a victory for the plaintiff: The next critic may be less inclined to speak out given the financial risks.

## Conflict of interest

The authors declare that they have no conflict of interest.
